# Tobacco companies are booming despite an economic depression

**DOI:** 10.1186/1617-9625-5-9

**Published:** 2009-06-15

**Authors:** Peisen He, Eiji Yano

**Affiliations:** 1 Department of Hygiene and Public Health, Teikyo University School of Medicine, Tokyo, Japan; 2 Department of Social Medicine, Harbin Medical University, Harbin, PR China

## Abstract

During the past year, an economic crisis has affected economies and life styles throughout the world. However, the three largest transnational tobacco companies – Philip Morris International, British American Tobacco, and Japan Tobacco – showed excellent returns during this period, reflecting more widespread indirect exposure to smoking.

## Letter

Since September of 2008, a serious global economic recession has affected many businesses and caused widespread unemployment. However, certain economic entities have continued to earn profits despite the difficulties affecting the rest of the world.

Philip Morris International (PMI), British American Tobacco (BAT), and Japan Tobacco (JT) constitute the three largest international manufacturers of tobacco products in the world, with 2007 market shares of 15.6% [1], 12.0% and 10.6% respectively [2].

PMI, separated from the Atria Group on March 28, 2008, is currently the largest transnational tobacco company [3], with products sold in approximately 160 countries [1]. In 2008, the cigarette sales of PMI totaled 869.7 billion, and its gross turnover was 63.64 billion dollars, representing increases of 2.5% and 15.2%, respectively, as compared to the previous year [3]. BAT is the second largest international tobacco company in the world, conducting business in more than 180 countries and areas. Its sales volume and gross turnover in 2008 were 715.0 billion units and 62.82 billion dollars, representing increases of 4.5% and 25.2%, respectively, as compared to 2007 [3]. The third largest tobacco company, JT, with tobacco products in more than 120 countries [2], sold 614.1 billion cigarettes in 2008, an increase of 10.8% over the previous year, which included 452.3 billion sold in the international market and 161.8 billion sold in the domestic market. The gross turnover of JT was 58.21 billion dollars, an increase of 10.2%, as compared to the previous year [3].

The sales records of these tobacco companies demonstrate that smokers not only continued to smoke but also actually increased their cigarette intake during this period of economic difficulty, despite the harm to everyone caused by exposure to this habit. Tobacco has already killed 100 million people in the 20th century, and 5.4 million deaths per year currently result from tobacco use. In the absence of urgent action, more than 8 million tobacco-related deaths are predicted for every year beginning in 2030, and approximately 1 billion tobacco-related deaths are predicted to occur during the 21st century [4]. We must intensify attention on the responsibility of tobacco companies for tobacco control.

## Competing interests

The authors declare that they have no competing interests.

## Authors' contributions

EY conceived the idea for the study. PH and EY wrote the manuscript. Both authors read and approved the final version of the manuscript.
